# Brewing method‐dependent changes of volatile aroma constituents of green tea (*Camellia sinensis* L.)

**DOI:** 10.1002/fsn3.4307

**Published:** 2024-07-16

**Authors:** Canan Göksu Sürücü, Aysu Tolun, Ozan Halisçelik, Nevzat Artık

**Affiliations:** ^1^ Plant‐Based Food Research Center, Field Crops Central Research Institute, Directorate General of Agricultural Research and Policies Ankara Türkiye; ^2^ Department of Food Engineering Ankara University Ankara Türkiye; ^3^ Core Unit Metabolomics, Berlin Institute of Health Charité University Berlin Germany

**Keywords:** aromatic volatile compounds, brewing, *Camellia sinensis* L., GC–MS, green tea, SPME

## Abstract

The determination of optimal levels of green tea amount and brewing time would have a crucial role in the accumulation of desired aromatic volatile compounds to meet worldwide market demand. Aroma is the most important factor influencing tea consumers' choices along with taste, price, and brand. This study aims to determine how the brewing time and amount of green tea affect the aroma profile of green tea infusion. The effect of the amount of Turkish green tea (5–10 g) and brewing time (5–60 min) on aromatic volatile compounds was evaluated using solid‐phase microextraction (SPME) and gas chromatography–mass spectrometry (GC–MS) technique. The SPME/GC–MS analysis identified 57 components in the aroma profile of green tea infusions including 13 esters, 12 alkanes, 7 unknowns, 6 ketones, 3 alcohols, 2 terpenes, 2 terpenoids, 1 alkaloid, 1 phenolic compound, 1 lactone, 1 pyrazine, and 1 norisoprenoid. The green tea amount and brewing time had significant effects on the number of chemical compounds. A total of 42, 47, and 36 aromatic volatile compounds were determined by brewing 5, 7.5, and 10 g of green tea. The most abundant constituents in green tea infusions were phytone, 2‐decenal, lauric acid, unknown 1, methoxy‐1‐methylethyl pyrazine, α‐ionone, β‐ionone, and diethyl phthalate (DEP). With this study, the aroma structures of green tea infusion have been revealed for the first time depending on the brewing time and quantity.

## Graphical abstract

With this study, the aroma structures of green tea infusion have been revealed for the first time depending on the brewing time and quantity. According to the results, the amount of brewed green tea had a considerable impact on the aroma composition. In addition, extending the brewing time significantly altered the distribution and quantity of aromatic components in green tea infusions.

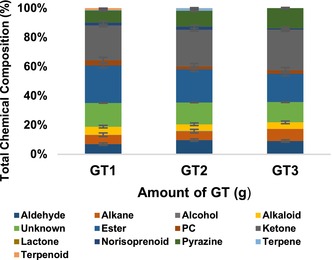

## 1 INTRODUCTION

Tea (*Camellia sinensis* L.) is an economically important plant and tea infusion is the second most widely consumed beverage worldwide after water. Green tea (GT) accounts for roughly 30% of the worldwide tea market in the tea industry (Ho et al., 2015; Li et al., 2024). Approximately 2 billion people drink tea throughout 170 countries and regions, while more than 60 countries cultivate the tea plant (Yin et al., 2022). There are different types of tea, which can be divided into six different categories based on sensory qualities and processing methods with specific flavor profiles. These include GT, white tea, black tea, dark tea, yellow tea, and oolong tea that differ in the degree of fermentation with the oxidation of catechins (flavan‐3‐ols) (Baldermann et al., 2014; Guo et al., 2019; Wang et al., 2018). GT is one of the least oxidized teas of all the teas. GT is an angiosperm of the dicot plant *Camellia sinensis*, which can retain green leaves throughout the year. This type of tea can be produced as an aromatic herbal beverage from unfermented leaves in a low‐cost unit operation with minimal processing (Anand et al., 2015).

The consumption of this fermented beverage has numerous health benefits, such as anti‐oxidation, anticancer, antiaging, anti‐inflammatory, and sterilization properties (Liao et al., 2020). In addition, GT is an efficient drink for the prevention of tooth decay and ulcers, the increase of bacterial flora in the intestine and bone density, the improvement in stomach diseases and disorders, the control of halitosis, and the protection against ultraviolet (UV) radiation‐related damages (Guo et al., 2019). GT's health benefits have made it increasingly popular, especially in East Asian countries.

The first step in drinking tea is typically to brew it. In general, making tea entails placing tea leaves in a cup and filling it with hot drinking water or using boiling water for brewing, and keeping the temperature at boiling level. Compounds in tea can leach depending on a number of brewing parameters, such as the ratio of tea to water, time, temperature, and quality of brewing water (Lee, Chambers, & Chambers IV, 2013; Lin et al., 2019; Zhang et al., 2017).

The aromatic volatile organic compounds (VOCs) are one of the most important quality parameters to increase the consumer preferences of tea. Since these compounds are naturally complex with low concentrations, sample preparation and extraction are so important to detect volatiles using analytical procedures, such as gas chromatography–mass spectrometry (GC–MS) (Qin et al., 2024; Zhang et al., 2017). Since the 1990s, thorough studies have been carried out to identify the primary odorants in tea, and current research on the subject is still ongoing (Cao et al., 2022). Volatile fractions in GT contain more than 600 aroma‐active compounds that give a variety of odor notes, such as green, floral, nutty, fruity meaty, potato‐like, popcorn‐like, metal‐like, straw‐like, and cucumber‐like. Pleasing green tea aromas are frequently characterized as tender, faint scent, orchid‐like, and chestnut‐like (Cao et al., 2022; Ho et al., 2015; Kumazawa & Masuda, 2002; Qin et al., 2024; Rigling et al., 2021). On the other hand, the dynamic variations in the aroma profile still are little known as a function of drying conditions under various temperature gradients and times. Although, studies on the aroma of green tea under various conditions have been published, to the best of our knowledge, no studies have been conducted on the effect of brewing time and amount of green tea on the aroma components in the infusion. Hence, the current study aimed to analyze the profile of VOCs of dissimilar amounts at different brewing times to understand the effect of infusion time and green tea amount on volatile aroma components of green tea infusions.

## 2 MATERIALS AND METHODS

### 2.1 Raw material

Fresh GT leaves were gathered from the Eastern Black Sea Region of Türkiye in 2020 and processed into GT at the General Directorate of Tea Enterprises (CAYKUR) Green Tea Factory.

### 2.2 Sample preparation

To analyze aroma compounds, samples were prepared by brewing various quantities of fine particles (215 ± 75 μm) of phenolics‐rich GTs (i.e., 5 g [GT1], 7.5 g [GT2], and 10 g [GT3]) for 12 times with 5 min increments (i.e., 5, 10, 15, 20, 25, 30, 35, 40, 45, 50, 55, and 60 min). The Turkish‐style brewing technique was utilized (Göksu Sürücü & Artık, 2022). This procedure was carried out in a porcelain teapot with 250 mL of distilled boiling water and it then remained at boiling temperature. The tea infusions were then rapidly filtered using a Whatman No. 1 filter paper under the vacuum. After preparing the green tea infusions, the test tubes were carefully closed and covered in Parafilm® around the edges to avoid aroma loss. Until further analysis, the samples were kept at −18°C and wrapped in aluminum foil.

### 2.3 SPME analysis

Solid‐phase microextraction was based on the method described by Feng et al. (2022) with modifications. In brief, 10 mL samples were taken and placed in 20 mL vials, and closed using the lids. After the addition of the internal standards (β‐myrcene, 2 μL and 5 mg/L) and sample equilibration (30°C, 30 min), the vials were vortexed for 2 s. A 50/30‐μm 2‐cm fiber (divinylbenzene/carbozen/polydimethylsiloxane (DVB/CAR/PDMS), Supelco, Bellefonte, PA, USA) was previously conditioned at 200°C for 20 min in GC–MS and attached to the vial at 55°C for 30 min. Lastly, the fiber was automatically injected into GC–MS, and analyses were performed (Göksu Sürücü, 2022).

### 2.4 GC–MS analysis of aroma components

The aroma analysis was performed using a GC–MS system (model AOC‐6000, Shimadzu, Tokyo, Japan) with the column of RTX‐5MS (30 m × 0.25 mm × 0.25 μm, Restek Co., Bellefonte, PA). The method described by Lau et al. (2018) with minor modifications depending on the nesting of peaks was used to identify and quantify VOCs. The oven temperature program was as follows: initially set at 40°C (isothermal for 3 min), gradually increased from 40 to 240°C with a rate of 4°C/min, and finally, isothermally kept at 240°C for 5 min. The following parameters were used for the analysis: 250°C injection temperature, 90.0 kPa pressure, 1.61 mL/min column flow rate, 20.7 mL/min total flow rate, and 1:10 partition coefficient. The mass detector was set in an ion mode (electron ionization (EI)) at an ionization voltage of 70 eV in the 35–450 amu (atomic mass unit) scan range for mass spectrum collection, event time was 0.3 ms, and the ion source temperature was 200°C. The retention index (RI) was calculated for individual constituents using retention times of the reference n‐alkanes (C7–C30, Merck Chemical Co., Darmstadt, Germany). The identification of compounds was performed based on their calculated RI indices compared to the Flavor and Fragrance Natural and Synthetic Compounds (FFNSC) library, computer matching with commercial mass spectral libraries (McLafferty et al., 1989; NIST, 2017), and the comparison of their mass spectra with those of an in‐house laboratory library. The relative intensity of each compound has been calculated as the ratio between the area of the specific molecule and the sum of the areas of all identified peaks (peak area normalization method) in the chromatogram (Selli et al., 2012).

### 2.5 Statistical analysis

The experiments were performed in duplicate and the data were subjected to analysis of variance (ANOVA). Mean values were compared by *t*‐test (least significant difference [LSD]) and considered significantly different at *p* ≤ 0.01. Different letters indicate statistically significant differences between samples.

## 3 RESULTS AND DISCUSSION

**FIGURE 1:**
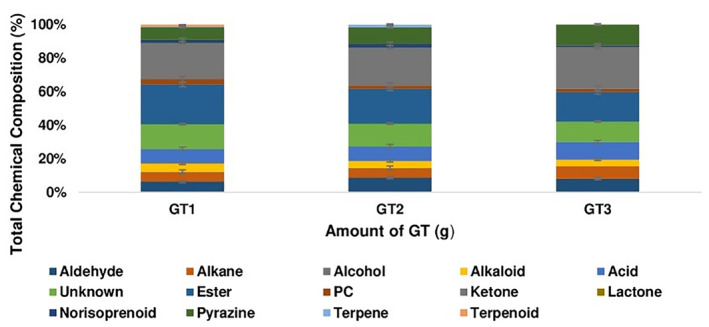
Changes in aroma composition of green tea infusions as a function of brewing amount (5, 7.5, and 10 g) for 5, 10, 15, 20, 25, 30, 35, 40, 45, 50, 55, and 60 min brewing, GT, Green tea; PC, phenolic compound. GT1, GT2, and GT3 indicate 5, 7.5, and 10 g samples, respectively.

**FIGURE 2:**
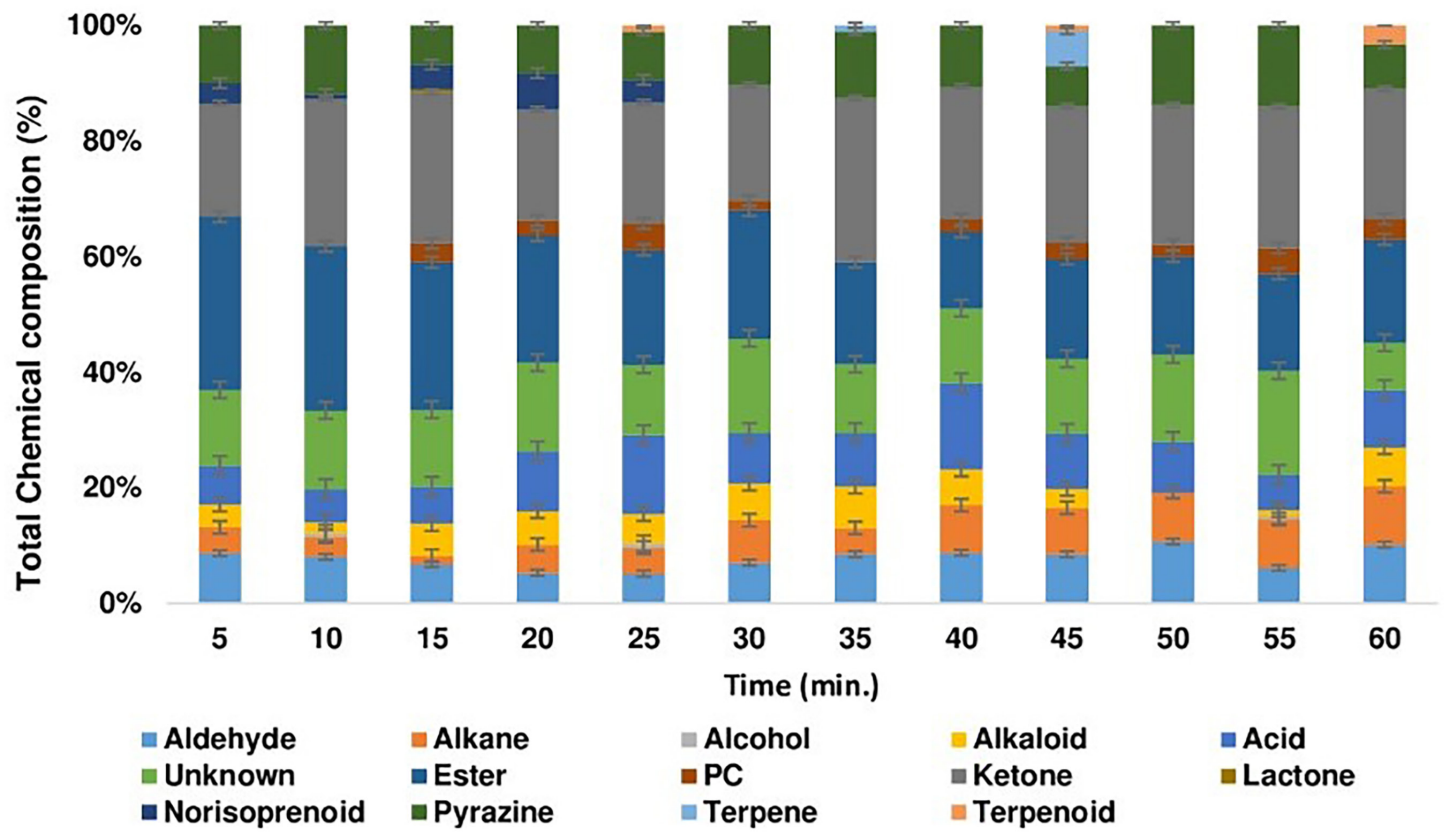
Changes in aroma composition of green tea infusions as a function of brewing time (5, 10, 15, 20, 25, 30, 35, 40, 45, 50, 55, and 60 min) for 5, 7.5, and 10 g amounts, respectively. PC, Phenolic compound.

Changes in the aroma composition of infusions as a function of brewing amount and time are shown in Figures 1 and 2, descriptions of aroma components of infusions are given in Table 1, and the volatile aroma components and relative contents are shown in Table 2.

**TABLE 1:** Description of aroma components of green tea infusions.

Components	CAS No.	Aroma description/type	Reference
Acids			
Lauric acid	143‐07‐7	Fatty odor	Burdock (2016) and FAO (2024)
Palmitic acid	57‐10‐3	Virtually odorless	Burdock (2016) and FAO (2024)
Nonanoic acid	112‐05‐0	Fatty, characteristic odor and a corresponding unpleasant taste, having a cheese, waxy flavor	Burdock (2016) and FAO (2024)
Alcohols			
3,7‐Dimethyl‐1‐octanol (Hydroxycitronellol)	106‐21‐8	Mild sweet odor reminiscent of rose and grape hyacinth	Burdock (2016) and FAO (2024)
Linalool	78‐70‐6	Typical pleasant floral odor, free from camphoraceous and terpenic notes	Burdock (2016) and FAO (2024)
Lauryl alcohol	112‐53‐8	Fatty odor	FAO (2024)
Aldehydes			
n‐Nonanal	124‐19‐6	Strong, fatty odor developing an orange and rose note on dilution. It has a fatty, citrus‐like flavor.	Burdock (2016)
Decanal	112‐31‐2	Floral‐orange odor	Burdock (2016) and FAO (2024)
cis‐8‐Undecenal	147159‐49‐7	Strong and pleasant aldehyde odor	TGSC (2022a)
2‐Undecenal	2463‐77‐6	Fresh, fruity, orange peel aroma	Burdock (2016) and FAO (2024)
2‐Octenal	2363‐89‐5	Fatty, green aroma, peculiar green‐leafy odor, orange, honey‐like, cognac‐like aroma	Burdock (2016) and FAO (2024)
Alkaloids			
Caffeine	58‐08‐2	Virtually odorless	Burdock (2016)
Alkanes			
Nonadecane	629‐92‐5	Chemical	NIH (2022a)
Heptadecane	629‐78‐7	Chemical	Guo et al. (2021)
Hexadecane	544‐76‐3	Chemical	Guo et al. (2021)
Eicosane	112‐95‐8	Chemical	NIH (2022b)
Octadecane	593‐45‐3	Chemical	Guo et al. (2021)
Pentacosane	629‐99‐2	Chemical	Guo et al. (2021)
Pentadecane	629‐62‐9	Chemical	Guo et al. (2021)
Tetradecane	629‐59‐4	Chemical	Guo et al. (2021)
Docosane	629‐97‐0	Chemical	Guo et al. (2021)
Neodene		Faint petroleum hydrocarbon odor	NIH (2022c)
Esters			
Isobornyl acetate	125‐12‐2	Camphoraceous, piney, balsamic aroma	Burdock (2016) and FAO (2024)
Diethyl phthalate	84‐66‐2	No pronounced odor, bitter, unpleasant taste	NIH, 2022d
Ethyl salicylate	118‐61‐6	Spicy, anisic, wintergreen‐like aroma	Burdock (2016) and FAO (2024)
Isopropyl myristate	110‐27‐0	Virtually odorless, very slightly fatty, but not rancid	Burdock (2016) and FAO (2024)
Methyl palmitate	112‐39‐0	Oily, waxy aroma	TGSC (2022b)
Esters			
Butyl laurate	106‐18‐3	Fruity, peanut odor	Burdock (2016) and FAO (2024)
ω‐Pentadecalactone	106‐02‐5	Extraordinarily persistent, musk‐like odor.	Burdock (2016) and FAO (2024)
Isoamyl benzoate	94‐46‐2	Mild, sweet, fruity‐like odor	Burdock (2016) and FAO (2024)
Tetrahydrofurfuryl butyrate	2217‐33‐6	Heavy sweet aroma reminiscent of apricot and pineapple	Burdock (2016) and FAO (2024)
Octyl octanoate	2306‐88‐9	Faint, fatty odor reminiscent of green tea and an oily, fruity, sweet, mildly green taste	Burdock (2016) and FAO (2024)
Decyl propionate	5454‐19‐3	Slightly fatty, aldehyde‐like odor reminiscent of cognac	Burdock (2016) and FAO (2024)
Methyl anthranilate	134‐20‐3	Grape‐like or orange aroma	Burdock (2016) and FAO (2024)
Di‐isobutyl phthalate	84‐69‐5	Slight ester odor	NIH (2022e)
Phenolic compounds			
Butylated hydroxytoluene	128‐37‐0	Very light, musty, occasionally cresylic‐type odor	Burdock (2016)
Ketones			
α‐Ionone	127‐41‐3	Warm, woody, violet‐floral odor	Burdock (2016) and FAO (2024)
β‐Ionone	79‐77‐6	Warm, woody, dry odor	Burdock (2016) and FAO (2024)
Phytone	502‐69‐2	Jasmine odor	Fanaro et al. (2011) and Dai et al. (2020)
3‐Methyl‐2‐(n‐pentanyl)‐2‐cyclopenten‐1‐one	1128‐08‐1	Fresh, fruity, jasmine odor with woody and herbal nuances	Burdock (2016) and FAO (2024)
6‐methyl‐5‐hepten‐2‐one	110‐93‐0	Strong, fatty, green, citrus‐like odor and bittersweet taste reminiscent of pear	Burdock (2016) and FAO (2024)
Geranyl acetone	3796‐70‐1	Fruity‐sweet, fruity‐flowering, pink, green magnolia	Lv et al. (2012) and Dai et al. (2020)
Lactones			
5‐Hydroxy‐2,4‐decadienoic acid δ‐lactone	27593‐23‐3	Mushroom, blue cheese lactone or dairy odor	Burdock (2016) and FAO (2024)
Monoterpen			
Pulegone	89‐82‐7	Herbaceous‐minty, resinous odor, pleasant odor, somewhat similar to peppermint and camphor	Burdock (2016) and FAO (2024)
Monoterpenoid			
Isopulegyl acetate	57576‐09‐7	Fresh, green‐minty, leafy, sweet fruity odor	Burdock (2016) and FAO (2024)
Norisoprenoid			
Theaspirane	36431‐72‐8	Fruity, woody, sweetish, and ionone‐like camphoraceous	Burdock (2016) and FAO (2024)
Sesquiterpenes			
α‐Cubebene	17699‐14‐8	Slight camphor odor	TGSC (2022c)
Sesquiterpenoids			
Davanone B	20482‐11‐5	Odorless	Guenther et al. (1967)
Nitrogen‐containing compounds			
Methoxy‐1‐methylethyl pyrazine	–	Nutty and roasted odor	Anand et al. (2015)

**TABLE 2:** The determined levels of aromatic volatile compounds present in GT infusions obtained during different brewing amounts and times (%).

Aroma components	Time (min)								
	5			10			15		
	GT1	GT2	GT3	GT1	GT2	GT3	GT1	GT2	GT3
n‐Nonanal	(1.81 ± 0.03)^ij^	–	(2.24 ± 0.01)^de^	–	–	(2.67 ± 0.03)^b^	–	(2.06 ± 0.03)^fg^	–
2‐Decenal	(6.93 ± 0.04)^cf^	(5.50 ± 0.04)^fj^	(5.21 ± 0.01)^gk^	(5.10 ± 0.01)^hk^	(7.97 ± 0.01)^bd^	(8.42 ± 0.03)^bd^	(7.27 ± 0.02)^be^	(6.81 ± 0.02)^dg^	(3.65 ± 0.01)^kl^
Isobornyl acetate	(2.37 ± 0.01)^b^	(2.82 ± 0.02)^a^	–	(1.64 ± 0.03)^c^	–	–	–	–	–
Unknown 1	(6.78 ± 0.01)^x^	(9.60 ± 0.03)^n^	(6.43 ± 0.02)^z^	(7.55 ± 0.04)^u^	(12.65 ± 0.01)^d^	(5.88 ± 0.04)^bl^	(11.55 ± 0.07)^f^	(6.25 ± 0.01)^al^	(7.02 ± 0.03)^w^
α‐Ionone	(2.72 ± 0.04)^no^	(3.46 ± 0.01)^jk^	(5.13 ± 0.01)^e^	(3.65 ± 0.02)^ij^	(5.82 ± 0.01)^d^	(9.17 ± 0.01)^b^	(11.97 ± 0.01)^a^	(5.72 ± 0.02)^d^	(5.79 ± 0.04)^d^
β‐Ionone	(7.75 ± 0.04)^p^	(9.45 ± 0.04)^l^	(12.94 ± 0.00)^b^	(7.39 ± 0.01)^rs^	(10.97 ± 0.02)^g^	(17.73 ± 0.04)^a^	–	(11.70 ± 0.08)e	(9.41 ± 0.01)l^m^
Methoxy‐1‐methylethyl pyrazine	(7.51 ± 0.01)^u^	(9.20 ± 0.14)^q^	(10.29 ± 0.03)^n^	(7.20 ± 0.03)^q^	(12.55 ± 0.02)^g^	(15.83 ± 0.02)^a^	–	(12.52 ± 0.02)^g^	(8.51 ± 0.02)^r^
Diethyl phthalate	(24.32 ± 0.03)^a^	(18.15 ± 0.21)j	(22.61 ± 0.03)^e^	(22.88 ± 0.03)^d^	(22.25 ± 0.03)^f^	(23.44 ± 0.03)^c^	(19.56 ± 0.03)^h^	(23.95 ± 0.01)^b^	(15.30 ± 0.01)^o^
Nonadecane	(2.87 ± 0.06)^g^	–	(2.78 ± 0.01)^h^	–	–	–	–	–	–
Lauric acid	(2.27 ± 0.01)^y^	(3.98 ± 0.01)^m^	(4.88 ± 0.05)^k^	(4.70 ± 0.03)^l^	(6.04 ± 0.02)^f^	(6.51 ± 0.01)^d^	(3.33 ± 0.02)^q^	(5.44 ± 0.01)^h^	(3.30 ± 0.02)^qr^
Ethyl salicylate	(2.54 ± 0.06)^f^	–	–	–	–	–	–	(3.47 ± 0.01)^c^	–
Isopropyl myristate	(7.07 ± 0.04)^b^	(3.05 ± 0.04)^c^	(2.14 ± 0.03)^e^	(4.66 ± 0.04)^c^	–	–	–	–	–
Caffeine	(4.10 ± 0.01)^s^	(2.22 ± 0.01)^w^	(4.41 ± 0.03)^qr^	(5.68 ± 0.01)^l^	–	–	(5.92 ± 0.04)^k^	(5.33 ± 0.04)^m^	(5.94 ± 0.02)^k^
Phytone	(6.78 ± 0.04)^t^	(5.91 ± 0.01)^u^	(7.41 ± 0.01)^rs^	(7.22 ± 0.03)^s^	(6.89 ± 0.01)^t^	(7.95p ± .01)^p^	(8.66 ± 0.01)^n^	(7.58 ± 0.03)^qr^	(7.69 ± 0.42)^q^
Theaspirane	(4.90 ± 0.02)^e^	(4.35 ± 0.02)^f^	–	(2.26 ± 0.01)^k^	–	–	(3.90 ± 0.01)^g^	(6.34 ± 0.02)^b^	(3.12 ± 0.01)^h^
Palmitic acid	(5.96 ± 0.04)^a^	–	–	–	–	–	–	–	–
Unknown 2	(3.27 ± 0.02)^a^	–	–	–	–	–	–	–	–
Decanal	(1.82 ± 0.02)^c^	–	–	–	–	–	–	–	(1.24 ± 0.03)^d^
Unknown 3	–	(2.13 ± 0.02)^d^	–	–	(2.55 ± 0.01)^a^	–	–	–	–
Heptadecane	–	(1.62 ± 0.04)^e^	(1.85 ± 0.01)^d^	–	–	–	–	–	–
Unknown 4	–	(4.30 ± 0.03)^c^	‐	–	(3.40 ± 0.03)^e^	–	(8.33 ± 0.01)^a^	‐	(3.25 ± 0.01)^f^
Hexadecane	(2.93 ± 0.04)^e^	–	‐	–	–	–	–	(3.05 ± 0.03)^d^	–
Methyl palmitate	–	(1.67 ± 0.01)^e^	(1.96 ± 0.04)^d^	(1.63 ± 0.03)^e^	(2.72 ± 0.03)^a^	–	–	–	–
Butyl laurate	–	(3.74 ± 0.03)^a^	–	–	–	–	–	–	–
ω‐Pentadecalactone	–	(1.63 ± 0.00)^b^	–	(1.97 ± 0.01)^a^	–	–	(2.01 ± 0.03)^a^	–	(1.76 ± 0.03)^b^
Nonanoic acid	–	–	(2.22 ± 0.01)^t^	–	–	–	–	–	(7.83 ± 0.01)^i^
Unknown 5	–	(5.01 ± 0.02)^j^	–	(6.42 ± 0.02)^d^	–	–	–	–	–
Lauryl alcohol	–	–	–	(1.82 ± 0.01)^a^	–	–	–	–	–
Eicosane	–	–	–	(3.87 ± 0.01)^e^	(4.27 ± 0.03)^c^	(2.43 ± 0.02)^g^	–	–	–
Unknown 6	–	–	–	(2.23 ± 0.03)^b^	–	–	–	–	(1.35 ± 0.03)^c^
Isoamyl benzoate	–	–	–	(1.96 ± 0.01)^g^	(1.99 ± 0.03)^g^	–	–	–	(2.64 ± 0.02)^de^
3‐Methyl‐2‐(n‐pentanyl)‐2‐cyclopenten‐1‐one	–	–	–	–	–	–	(11.63 ± 0.04)^a^	–	–
5‐Hydroxy‐2,4‐decadienoic acid δ‐lactone	–	–	–	–	–	–	–	–	(1.14 ± 0.03)^a^
Unknown 7	–	–	–	–	–	–	–	–	(3.67 ± 0.03)^a^
6‐Methyl‐5‐hepten‐2‐one	–	–	–	–	–	–	–	–	(1.47 ± 0.01)^a^
Octadecane	–	–	–	–	–	–	–	–	(1.57 ± 0.02)^j^
Butylated hydroxytoluene	–	–	–	–	–	–	–	–	(10.21 ± 0.04)^b^
Tetrahydrofurfuryl butyrate	–	–	–	–	–	–	–	(10.47 ± 0.00)^a^	–

Aroma components	Time (min)								
	20			25			30		
	GT1	GT2	GT3	GT1	GT2	GT3	GT1	GT2	GT3
n‐Nonanal	–	(0.77 ± 0.02)^l^	–	–	(0.90 ± 0.02)^l^	(2.01 ± 0.00)^gh^	–	(2.00 ± 0.01)^g^	–
2‐Decenal	(5.46 ± 0.01)^hk^	(4.17 ± 0.01)^jk^	(4.43 ± 0.02)^ik^	(4.95 ± 0.02)^hk^	(3.75 ± 0.01)^kl^	(5.01 ± 0.04)^hk^	(4.71 ± 0.02)^hk^	(6.00 ± 0.02)^ei^	(6.88 ± 0.02)^df^
Unknown 1	(9.95 ± 0.01)^l^	(6.49 ± 0.02)^z^	(7.37 ± 0.01)^v^	(10.33 ± 0.04)^k^	(7.65 ± 0.01)^t^	(8.23 ± 0.02)^r^	(10.98 ± 0.03)^h^	(11.82 ± 0.01)^e^	(5.03 ± 0.02)^cl^
α‐Ionone	(3.37 ± 0.04)^jl^	(3.54 ± 0.03)^j^	(3.95 ± 0.03)^hi^	(2.51 ± 0.01)^op^	(3.92 ± 0.03)^hi^	(4.58 ± 0.01)^f^	(3.22 ± 0.02)^km^	(2.22 ± 0.03)^pq^	(3.92 ± 0.01)^hi^
β‐Ionone	(7.15 ± 0.02)^t^	(6.24 ± 0.02)^w^	(8.94 ± 0.01)^n^	(6.44 ± 0.04)^v^	(7.20 ± 0.01)^st^	(9.83 ± 0.01)^k^	(7.66 ± 0.01)^pq^	(6.82 ± 0.03)^u^	(9.83 ± 0.01)^k^
Methoxy‐1‐methylethyl pyrazine	(8.14 ± 0.01)^s^	(6.15 ± 0.01)^y^	(10.08 ± 0.03)^o^	(6.63 ± 0.01)^x^	(7.01 ± 0.03)^w^	(11.45 ± 0.00)^k^	(9.64 ± 0.03)^p^	(9.57 ± 0.01)^p^	(11.73 ± 0.01)^j^
Diethyl phthalate	(17.36 ± 0.04)^l^	(11.76 ± 0.24)^w^	(17.37 ± 0.04)^l^	(12.90 ± 0.02)^t^	(11.84 ± 0.02)^w^	(14.27 ± 0.01)^q^	(21.67 ± 0.39)^g^	(14.64 ± 0.02)^p^	(18.00 ± 0.03)^jk^
Nonadecane	(4.84 ± 0.02)^c^	–	–	–	–	(4.43 ± 0.01)^f^	–	–	(4.42 ± 0.03)^e^
Lauric acid	–	(5.27 ± 0.01)^i^	(4.71 ± 0.01)^l^	(4.83 ± 0.11)^k^	(3.68 ± 0.04)^n^	(3.58 ± 0.01)^o^	(2.55 ± 0.03)^w^	(4.98 ± 0.02)^j^	(3.58 ± 0.02)^o^
Ethyl salicylate	–	(4.54 ± 0.04)^b^	(2.66 ± 0.01)^e^	(3.08 ± 0.01)^d^	(2.33 ± 0.03)^g^	–	–	–	–
Isopropyl myristate	–	–	–	–	–	–	–	–	(8.15 ± 0.01)^a^
Caffeine	(8.04 ± 0.03)^c^	(5.14 ± 0.02)^n^	(3.96 ± 0.03)^t^	(4.81 ± 0.01)^p^	(4.14 ± 0.04)^s^	(6.55 ± 0.01)^g^	(7.20 ± 0.01)^e^	(5.64 ± 0.01)^l^	(6.42 ± 0.03)^h^
Phytone	(8.27 ± 0.04)^o^	(5.67 ± 0.02)^v^	(8.12 ± 0.01)^op^	(8.12 ± 0.02)^op^	(8.02 ± 0.01)^p^	(9.55 ± 0.02)^ij^	(9.86 ± 0.01 g)^h^	(9.13 ± 0.01)^lm^	(8.25 ± 0.04)^o^
Theaspirane	(3.64 ± 0.03)^a^	(9.41 ± 0.01)^a^	(5.43 ± 0.03)^d^	(5.96 ± 0.02)^c^	(3.52 ± 0.01)^i^	(2.19 ± 0.01)^l^	–	–	–
Decanal	–	–	(1.12 ± 0.01)^e^	–	–	–	–	–	(1.75 ± 0.03)^c^
Unknown 3	(2.17 ± 0.04)^d^	(1.34 ± 0.01)^i^	–	–	(1.46 ± 0.02)^h^	‐	(2.03 ± 0.01)^e^	(1.73 ± 0.03)^f^	–
Heptadecane	–	–	–	–	‐	(1.42 ± 0.03)^f^	(2.04 ± 0.04)^c^	‐	–
Unknown 4	(2.61 ± 0.04)^h^	–	–	–	(3.65 ± 0.04)^d^	–	–	(5.15 ± 0.02)^b^	–
Hexadecane	–	(2.35 ± 0.03)^g^	–	–	–	–	–	–	–
Methyl palmitate	–	(2.63 ± 0.03)^b^	(2.17 ± 0.01)^c^	(1.51 ± 0.01)^f^	–	–	–	–	–
Butyl laurate	–	–	–	–	–	–	–	–	–
ω‐Pentadecalactone	–	–	–	–	–	–	–	–	–
Nonanoic acid	(9.83 ± 0.03)^c^	(4.31 ± 0.04)^p^	(6.52 ± 0.03)^j^	(8.63 ± 0.01)^f^	(8.53 ± 0.01)^g^	(11.95 ± 0.03)^p^	(4.50 ± 0.02)^o^	(6.54 ± 0.04)^j^	(4.25 ± 0.03)^gj^
Unknown 5	(7.96 ± 0.01)^a^	–	(6.28 ± 0.03)^e^	–	‐	(5.53 ± 0.03)^f^	(7.21 ± 0.04)^b^	–	–
Lauryl alcohol	–	–	–	–	(1.24 ± 0.04)^b^	–	‐	–	–
Eicosane	–	–	(4.03 ± 0.01)^d^	–	(2.94 ± 0.03)^f^	–	(1.36 ± 0.02)^h^	–	–
Unknown 6	–	(1.05 ± 0.01)^d^	–	–	–	–	–	–	–
Isoamyl benzoate	–	(4.54 ± 0.04)^a^	–	(2.24 ± 0.01)^f^	(2.59 ± 0.02)^e^	–	‐	(2.67 ± 0.02)^d^	–
Octadecane	–	–	–	(3.30 ± 0.03)^h^	–	–	(4.76 ± 0.01)^e^	(4.22 ± 0.02)^g^	(1.35 ± 0.01)^k^
Butylated hydroxytoluene	–	(7.71 ± 0.02)^c^	–	(5.31 ± 0.02)^f^	(5.73 ± 0.02)^e^	–	–	–	(4.94 ± 0.02)^g^
Geranyl acetone	(1.65 ± 0.03)^a^	–	–	–	–	–	–	–	–
Linalool	–	–	–	–	(1.01 ± 0.01)^a^	‐	–	–	–
Tetrahydrofurfuryl butyrate	–	(1.90 ± 0.02)^d^	–	(4.92 ± 0.02)^b^	(2.76 ± 0.01)^c^	–	–	–	–
Pentacosane	–	(1.82 ± 0.03)^a^	–	–	–	–	–	–	–
Pentadecane	–	–	(1.41 ± 0.01)^f^	–	–	–	(2.35 ± 0.04)^b^	(1.67 ± 0.02)^e^	–
Isopulegyl acetate	–	–	–	(1.78 ± 0.02)^a^	(1.61 ± 0.03)^b^	–	–	–	–
Tetradecane	–	–	–	–	(0.97 ± 0.01)^d^	–	–	–	–
Docosane	–	–	–	–	(1.47 ± 0.001)^a^	–	–	–	–
Decyl propionate	–	–	–	–	–	–	–	(1.67 ± 0.02)^a^	–

Aroma components	Time (min)								
	35			40			45		
	GT1	GT2	GT3	GT1	GT2	GT3	GT1	GT2	GT3
n‐Nonanal	(1.67 ± 0.03)^j^	–	–	(2.35 ± 0.07)^ce^	(1.80 ± 0.14)^ij^	–	–	(2.37 ± 0.01)^cd^	(2.79 ± 0.01)^ab^
2‐Decenal	(6.10 ± 0.02)^eh^	(7.04 ± 0.01)^cf^	(7.34 ± 0.03)^be^	(7.53 ± 0.02)^be^	(7.75 ± 0.04)^bd^	(5.16 ± 0.03)^hk^	(2.33 ± 0.02)^l^	(11.75 ± 0.01)^a^	(4.82 ± 0.51)^hk^
Unknown 1	(9.72 ± 0.03)^m^	‐	(8.33 ± 0.02)^q^	(5.85 ± 0.01)^bl^	(8.25 ± 0.02)^r^	(10.82 ± 0.02)^i^	(11.23 ± 0.04)^g^	(6.68 ± 0.03)^y^	(9.06 ± 0.03)^o^
α‐Ionone	(3.91 ± 0.01)^hi^	(3.66 ± 0.01)^ij^	(5.06 ± 0.53)^e^	(3.65 ± 0.02)^ij^	(4.43 ± 0.01)^fg^	(5.16 ± 0.00)^e^	(2.07 ± 0.01)^qr^	(5.32 ± 0.04)^e^	(3.11 ± 0.02)^lm^
β‐Ionone	(7.44 ± 0.01)^r^	(10.70 ± 0.08)^h^	(12.34 ± 0.04)^c^	(7.47 ± 0.01)^qr^	(10.92 ± 0.03)^g^	(9.38 ± 0.01)^lm^	(4.90 ± 0.01)^x^	(12.13 ± 0.03)^d^	(10.34 ± 0.00)^i^
Methoxy‐1‐methylethyl pyrazine	(7.67 ± 0.02)^t^	(11.23 ± 0.01)^l^	(14.01 ± 0.03)^d^	(9.62 ± 0.01)^p^	(10.46 ± 0.02)^m^	(11.95 ± 0.01)^l^	(7.14 ± 0.01)^v^	–	(12.44 ± 0.01)^gh^
Diethyl phthalate	(19.17 ± 0.01)^i^	(12.23 ± 0.01^v^	(14.54 ± 0.03)^p^	(16.62 ± 0.01)^m^	(12.24 ± 0.02)^v^	(11.15 ± 0.01)^x^	(13.34 ± 0.03)^s^	(17.82 ± 0.01)^k^	(13.87 ± 0.02)^r^
Nonadecane	–	–	–	–	(4.74 ± 0.02)^d^	–	–	–	–
Lauric acid	(4.97 ± 0.02)^j^	(3.54 ± 0.04)^o^	(2.67 ± 0.04)^u^	(5.75 ± 0.03)^g^	(3.52 ± 0.01)^o^	(5.35 ± 0.02)^i^	(3.24 ± 0.01)^rs^	(6.76 ± 0.01)^e^	(3.17 ± 0.01)^s^
Isopropyl myristate	(1.42 ± 0.01)^f^	–	–	–	–	–	–	–	–
Caffeine	(3.76 ± 0.02)^u^	10.54 ± 0.03)^a^	(6.33 ± 0.01)^l^	(4.45 ± 0.01)^q^	(8.35 ± 0.00)^b^	(6.06 ± 0.04)^j^	(4.91 ± 0.04)^o^	–	–
Phytone	(9.74 ± 0.01)^hi^	(12.62 ± .03)^ab^	(12.78 ± 0.03)^a^	(12.53 ± 0.04)^b^	(10.54 ± 0.04)^b^	(9.37 ± 0.01)^jk^	(8.73 ± 0.01)^n^	(9.01 ± 0.02)^m^	(10.16 ± 0.01)^ef^
Decanal	–	(2.14 ± 0.01)^a^	–	–	–	(2.03 ± 0.02)^b^	‐	–	–
Unknown 3	–	–	–	–	–	(1.61 ± 0.01)^g^	(2.33 ± 0.03)^c^	–	–
Heptadecane	(3.86 ± 0.01)^a^	–	(2.16 ± 0.03)^b^	–	–	–	‐	–	–
Unknown 4	–	–	–	–	(2.27 ± 0.04)^i^	–	(4.37 ± 0.01)^c^	–	–
Hexadecane	–	–	–	–	–	–	(4.76 ± 0.01)^b^	–	–
Nonanoic acid	(2.45 ± 0.03)^s^	(6.22 ± 0.04)^k^	(6.53 ± 0.02)^j^	(9.30 ± 0.03)^d^	(9.85 ± 0.02)^c^	(11.55 ± 0.01)^b^	(8.36 ± 0.01)^h^	–	(5.43 ± 0.04)^m^
Unknown 5	(5.32 ± 0.02)^h^	(6.65 ± 0.04)^c^	(3.86 ± 0.04)^l^	–	(5.14 ± 0.01)^i^	(5.35 ± 0.02)^h^	–	(2.99 ± 0.03)^n^	–
Lauryl alcohol	–	–	–	–	–	–	–	–	–
Eicosane	–	–	(4.73 ± 0.03)^b^	(7.83 ± 0.03)^a^	–	–	–	–	–
Isoamyl benzoate	–	–	–	–	–	–	(3.79 ± 0.03)^b^	–	–
Octadecane	–	–	–	–	–	(5.02 ± 0.01)^d^	‐	(1.72 ± 0.03)^i^	(6.52 ± 0.02)^b^
Butylated hydroxytoluene	–	–	–	(6.88 ± 0.03)^d^	–	–	(4.84 ± 0.02)^h^	–	(3.23 ± 0.03)^j^
Pentadecane	–	–	–	–	–	(2.14 ± 0.01)^d^	(1.75 ± 0.02)^e^	–	(2.50 ± 0.04)^a^
Isopulegyl acetate	–	–	–	–	–	–	(1.61 ± 0.01)^b^	–	–
Tetradecane	–	–	–	–	–	–	–	–	(2.17 ± 0.01)^b^
Octyl octanoate	–	(3.00 ± 0.01)^a^	–	–	–	–	–	–	–
α‐Cubebene	(2.90 ± 0.01)^a^	–	–	–	–	–	–	–	–
Pulegone	–	–	–	–	–	–	–	(17.14 ± 0.02)^a^	–
Neodene	–	–	–	–	–	–	–	–	(1.75 ± 0.01)^a^
cis‐8‐Undecenal	–	–	–	(2.35 ± 0.04)^b^	(1.71 ± 0.03)^d^	(1.16 ± 0.01)^f^	–	(3.23 ± 0.00)^a^	–

Aroma components	Time (min)								
	50			55			60		
	GT1	GT2	GT3	GT1	GT2	GT3	GT1	GT2	GT3
n‐Nonanal	(1.81 ± 0.01)^ij^	(2.20 ± 0.14)^ef^	(2.87 ± 0.03)^a^	–	–	(2.45 ± 0.01)^c^	(1.45 ± 0.01)^k^	(1.83 ± 0.03)^hi^	(2.80 ± 0.04)^ab^
2‐Decenal	(8.77 ± 0.01)^b^	(7.26 ± 0.01)^be^	(8.16 ± 0.01)^bd^	–	(7.34 ± 0.00)^be^	(8.54 ± 0.01)^bc^	(4.45 ± 0.01)^ij^	(7.19 ± 0.03)^be^	(6.92 ± 0.03)^df^
Isobornyl acetate	–	–	–	–	–	–	–	–	–
Unknown 1	(13.95 ± 0.02)^c^	(10.46 ± 0.01)^j^	(10.97 ± 0.00)^h^	(7.77 ± 0.01)^s^	(17.45 ± 0.04)^a^	(14.67 ± 0.01)^b^	‐	‐	(8.62 ± 0.01)^p^
α‐Ionone	‐	(5.21 ± 0.01)^e^	(3.09 ± 0.01)^lm^	(6.36 ± 0.01)^c^	(1.88 ± 0.04)^r^	(3.65 ± 0.01)^ij^	(3.09 ± 0.03)^lm^	(4.13 ± 0.01)^gh^	(2.92 ± 0.03)^mn^
β‐Ionone	(7.97 ± 0.03)^o^	(10.67 ± 0.01)^h^	(10.10 ± 0.02)^j^	(12.42 ± 0.01)^c^	(7.84 ± 0.04o)^p^	(10.12 ± 0.01)^j^	(7.21 ± 0.02)^st^	(9.23 ± 0.01)^m^	(9.67 ± 0.029)^k^
Methoxy‐1‐methylethyl pyrazine	(12.40 ± 0.14)^h^	(13.04 ± 0.03)^f^	(14.55 ± 0.03)^c^	(13.02 ± 0.01)^f^	(13.43 ± 0.02)^e^	(15.34 ± 0.00)b	–	(9.64 ± 0.03)^p^	(11.73 ± 0.02)^j^
Diethyl phthalate	(22.60 ± 0.01)^e^	(13.79 ± 0.01)^r^	(12.56 ± 0.04)^u^	(17.45 ± 0.01)^l^	(15.81 ± 0.00)^n^	(9.46 ± 0.02)^y^	(15.62 ± 0.03)^n^	–	(13.48 ± 0.01)^s^
Nonadecane	–	(5.84 ± 0.03)^b^	(6.26 ± 0.02)^a^	–	–	–	–	(4.41 ± 0.01)^e^	(2.22 ± 0.02)^j^
Lauric acid	(2.32 ± 0.03)^yx^	(7.13 ± 0.03)^b^	(6.27 ± 0.01)^e^	(3.41 ± 0.01)^p^	(2.36 ± 0.01)^x^	(8.19 ± 0.00)^a^	(2.46 ± 0.03)^w^	(3.27 ± 0.03)^qr^	(3.04 ± 0.02)^t^
Ethyl salicylate	–	–	–	–	(7.42 ± 0.01)^a^	–	–	–	–
Caffeine	(3.20 ± 0.02)^v^	–	–	(6.79 ± 0.02)^f^	(7.74 ± 0.04)^d^	(4.37 ± 0.01)^r^	–	–	–
Phytone	(10.17 ± 0.02)^ef^	(12.56 ± 0.02)^b^	(10.12 ± 0.03)^ef^	(10.76 ± 0.03)^c^	(10.12 ± 0.01)^fg^	(10.25 ± 0.02)^e^	(8.56 ± 0.03)^n^	(8.64 ± 0.01)^n^	(9.24 ± 0.01)^kl^
Unknown 3	–	–	(2.47 ± 0.01)^b^	–	(2.57 ± 0.01)^a^	(1.63 ± 0.01)^g^	–	–	–
Heptadecane	–	–	–	–	–	–	–	–	–
Unknown 4	(2.77 ± 0.02)^g^	–	–	–	–	(2.30 ± 0.01)^i^	(1.76 ± 0.01)^j^	–	(2.81 ± 0.03)^g^
Hexadecane	(6.56 ± 0.03)^a^	–	–	–	–	(2.46 ± 0.03)^f^	(4.54 ± 0.04)^c^	(1.42 ± 0.01)^i^	–
Nonanoic acid	(5.53 ± 0.01)^l^	–	(3.93 ± 0.02)^q^	(2.73 ± 0.02)^r^	–	(1.37 ± 0.01)^u^	(4.43 ± 0.03)^o^	(9.22 ± 0.02)^e^	(5.12 ± 0.02)^n^
Unknown 5	(3.21 ± 0.01)^m^	–	–	–	(1.85 ± 0.03)^p^	(2.66 ± 0.03)^o^	–	(4.75 ± 0.01)^k^	–
Unknown 6	–	–	–	–	–	(2.85 ± 0.03)^a^	–	–	–
Octadecane	–	(3.55 ± 0.04)^g^	–	–	(7.83 ± 0.03)^a^	–	–	–	(6.24 ± 0.04)^c^
Butylated hydroxytoluene	–	(3.58 ± 0.04)^i^	(2.38 ± 0.01)^l^	(13.30 ± 0.01)^a^	‐	–	(6.90 ± 0.03)^d^	–	(3.03 ± 0.01)^k^
Pentadecane	–	–	(2.24 ± 0.03)^c^	‐	‐	–	(2.29 ± 0.02)^bc^	–	(2.43 ± 0.03)^a^
Tetradecane	–	–	(2.14 ± 0.02)^b^	‐	‐	(1.52 ± 0.02)^c^	–	(0.94 ± 0.01)^d^	(2.12 ± 0.03)^b^
Decyl propionate	–	–	(0.29 ± 0.00)^b^	‐	‐	‐	–	–	–
3,7‐Dimethyl‐1‐octanol	–	–	–	–	(1.70 ± 0.01)^a^	‐	–	–	–
Davanone B	–	–	–	–	–	–	(9.12 ± 0.01)^a^	–	–
cis‐8‐Undecenal	(2.07 ± 0.02)^c^	–	(2.00 ± 0.03)^c^	–	–	(1.54 ± 0.03)^e^	‐	–	–
2‐Undecenal	–	–	–	–	–	–	–	(1.71 ± 0.04)^a^	–
Methyl anthranilate	–	–	–	–	–	–	–	(11.27 ± 0.02)^a^	–
Di‐isobutyl phthalate	–	–	–	–	–	–	(4.60 ± 0.04)^a^	(2.02 ± 0.02)^b^	–
2‐Octenal	–	–	–	–	–	–	–	(1.94 ± 0.03)^a^	–

*Note*: Results are expressed as mean value±standard deviation (*n* = 2). Different letters in superscript within rows show significant differences (*p* ≤ .01); GT1, GT2, and GT3 indicate 5, 7.5, and 10 g samples, respectively; − indicates not detected aroma component.

### 3.1 Aroma profile of infusions

The SPME/GC–MS analysis revealed the presence of 57 different aromatic volatiles in the infusions. A total of 42, 47, and 36 aroma components were determined by brewing GT1, GT2, and GT3, respectively. However, seven volatile constituents found in GT infusions were unknown because they could not be detected in the FFNSC library. While the brewing time was less than 40 min in GT infusions, citrus‐like, woody, camphor‐like, fruity, floral, waxy, and oily aromas were observed in the infusion. The aroma profile changed with increasing brewing time. Resinous, cognac‐reminiscent, orange‐flavored, aldehyde‐like fragrant, and bitter taste‐forming compounds were found in the infusion (Table 1).

The number of aroma components was made to be different by changing the amount of brewed GT and the brewing time. The highest aroma components obtained for GT1, GT2, and GT3 were 19 (5–10 min), 24 (25 min), and 21 (15 min) units, respectively. On the other hand, the least aroma components obtained for GT1, GT2, and GT3 were 10 (55 min), 12 (35, 45–50 min), and 10 (10 min) units, respectively (Table 2). Among the identified 57 aroma components, there were 13 esters, 12 alkanes, 7 unknowns, 6 ketones, 3 alcohols, 2 terpenes, 2 terpenoids, 1 alkaloid, 1 phenolic compound, 1 lactone, 1 pyrazine, and 1 norisoprenoid (Figures 1 and 2). When GT infusions were examined in terms of time and amount, it was observed that there were differences in the distribution of aroma components in the infusions. The composition of GT1 aroma consisted of higher levels of alkaloid, ester, phenolic compound, and terpenoid; GT2 aroma consisted of higher levels of aldehyde, alcohol, norisoprenoid, and terpene; and GT3 aroma consisted of higher levels of alkane, acid, ketone, lactone, and pyrazine. Moreover, in terms of amount, alcohol, terpene, and terpenoid compounds were not found in GT3, while lactone was found only in GT3 (Table 2; Figure 1). In terms of time, terpene was observed only in brewing for 35 and 45 min, while alkaloid compounds were not found only in brewing for 60 min (Table 2; Figure 2).

### 3.2 Aroma components distribution of GT infusions

The most common compounds in GT infusions were phytone, 2‐decenal, lauric acid, α‐ionone, β‐ionone, methoxy‐1‐methylethyl pyrazine, unknown 1, and diethyl phthalate (Table 2). The only aroma component detected in all infusions was phytone, which has a jasmine scent and a warm floral aroma. The highest (12.78%) and lowest (5.67%) values of phytone were obtained by infusing GT2 for 35 min and GT2 for 20 min, respectively. The component of 2‐decenal was detected in 35 of 36 infusions (excluding GT1, 55 min). The highest (11.75%) and lowest (3.65%) values of this constituent were obtained by infusing GT2 for 45 min and GT3 for 15 min of brewing. Lauric acid was detected in 34 of 36 infusions (excluding GT1 for 20 min and GT2 for 35 min). The highest (8.19%) value was determined by infusing GT3 for 55 min, while the lowest value (2.27%) was detected by infusing GT1 for 5 min. Likewise, the unidentified aroma component in the FFNSC library named “unknown 1” was detected in 33 of 36 infusions (excluding GT2 for 35 min, and GT1 and GT2 for 60 min). The maximum (14.67%) and minimum (5.03%) values of unknown 1 were determined by infusing GT3 for 55 min and GT3 for 30 min. Methoxy‐1‐methylethyl pyrazine was detected in 33 of 36 infusions (excluding GT1 for 15 min or 60 min and GT2 for 45 min). The highest (11.97%) value of this component was determined by infusing GT1 for 15 min, while the lowest value (1.88%) was found in GT infusions obtained by infusing GT2 for 55 min. α‐Ionone was also detected in 35 of 36 infusions (excluding GT1 for 50 min). The highest (11.97%) and lowest (1.88%) values of α‐ionone were assessed in GT infusions obtained by infusing GT1 for 15 min and GT2 for 55 min, respectively. Besides, β‐ionone was detected in 35 of 36 infusions (excluding GT1 for 15 min). The maximum (17.73%) and minimum (7.21%) amounts of β‐ionone were detected in GT3 for 15 min and GT1 for 60 min, respectively. The presence of phytone (Dai et al., 2020; Fanaro et al., 2011;Wang, Sun, et al., 2021; Zhu et al., 2021), 2‐decenal (Burdock, 2016; Fanaro et al., 2011), lauric acid (Burdock, 2016; Ravichandran & Parthiban, 2000), methoxy‐1‐methylethyl pyrazine (Wang, Sun, et al., 2021; Zhu et al., 2021), α‐ionone (Ağca et al., 2020; Dai et al., 2020), and β‐ionone (Dai et al., 2019; Fanaro et al., 2011; Zhu et al., 2021) in GT infusions was also found in the literature.

In GT infusions, 20 aroma components were detected in only one infusion including palmitic acid (GT1 for 5 min, 5.96%), unknown 2 (GT1 for 5 min, 3.27%), butyl laurate (GT2 for 5 min, 3.74%), 3‐methyl‐2‐(n‐pentanyl)‐2‐cyclopenten‐1‐one (dihydrojasmone) (GT1 for 15 min, 11.63%), 5‐hydroxy‐2,4‐decadienoic acid δ‐lactone (GT3 for 15 min, 1.14%), unknown 7 (GT3 for 20 min, 3.67%), 6‐methyl‐5‐hepten‐2‐one (GT3 for 20 min, 1.47%), geranyl acetone (GT1 for 20 min, 1.65%), linalool (GT2 for 20 min, 1.01%), pentacosane (GT2 for 20 min, 1.82%), docosane (GT2 for 25 min, 1.47%), octyl octanoate (GT1 for 35 min, 3.0%), α‐cubebene (GT1 for 35 min 2.90%), pulegone (GT2 for 45 min 17.15%), neodene (GT3 for 45 min, 1.75%), 3,7‐dimethyl‐1‐octanol (hydroxycitronellol) (GT2 for 55 min, 1.70%), davanone B (GT1 for 60 min, 9.12%), 2‐undecenal (GT2 for 60 min, 1.71%), methyl anthranilate (GT2 for 60 min, 11.27%), and 2‐octenal (GT3 for 60 min, 1.94%). Apart from the most and least abundant flavor components, 30 other ones were detected in GT infusions, including: n‐nonanal, theaspirane, isobornyl acetate, unknown 3, heptadecane, decanal, unknown 4, nonadecane, isopropyl myristate, caffeine, ethyl salicylate, hexadecane, methyl palmitate, decyl propionate, nonanoic acid, unknown 5, eicosane, tetradecane, unknown 6, isoamyl benzoate, 6‐methyl‐5‐hepten‐2‐one, lauryl alcohol, butylated hydroxytoluene, tetrahydrofurfuryl butyrate, ω‐pentadecalactone, pentadecane, isopulegyl acetate, cis‐8‐undecanal, diisobutyl phthalate (DiBP), and octadecane (Göksu Sürücü, 2022).

Dai et al. (2019) reported that the main components of tea infusion were heptanal, hexanal, limonene, benzaldehyde, octanal, nonanal, methyl salicylate, β‐ionone, geranyl acetone, geraniol, decanal, linalool, nerolidol, and linalool oxides. Wang et al. (2016) also analyzed VOCs present in the famous Biluchun GTs grown in China. However, only 9 of the 67 aroma components detected in their study were the same as those detected in the present study. These components and their ratios are linalool (17.73%–30.63%), tetradecane (0.82%–2.35%), geranyl acetone (0.86%–4.03%), α‐ionone (0.29%–1.27%), β‐ionone (1.94%–8.98%), hexadecane (2.94%–4.17%), pentadecane (0.77%–2.06%), nonadecane (0%–0.58%), and heptadecane (1.63%–4.53%). In our study, 1.01% linalool was detected after brewing GT2 for 25 min, which is considerably lower than the amount reported by Wang et al. (2016), while much higher amounts of α‐ionone (1.88%–11.97%), β‐ionone (7.21%–17.73%), and nonadecane (2.78%–6.26%) were found in our study. The amounts of other aroma components are compatible with those mentioned in our study. Ağca et al. (2020) analyzed the VOCs collected from Northern Anatolia Region of Türkiye and detected trans‐2‐hexenal (3.9%), cis‐3‐hexenal (0.95%), n‐hexanal (0.31%), pentanol (1.19%), 1‐pentene‐3‐ol (0.49%), n‐hexanol (2.45%), 2,6,6‐trimethyl‐2‐hydroxycyclohexanone (7.06%), cis‐3‐hexenol (3.37%), β‐ionone (2.45%), n‐nonanal (6.66%), furfural (1.15%), n‐octanol (8.55%), trans‐2‐hexenol (4.63%), decanal (8.02%), nonanol (0.62%), cis‐3‐hexenyl hexanoate (11.26%), α‐ionone (3.72%), phenylacetaldehyde (5.40%), benzyl acetate (1.32%), geraniol (0.62%), and geranyl acetone (2.45%). Although we used GT grown in the same region in our study, β‐ionone (7.21%–17.73%), n‐nonanal (1.67%–2.87%), decanal (1.12%–2.24%), geranyl acetone (0%–1.65%), and α‐ionone (1.88%–11.97%) were detected and other compounds were not found (Göksu Sürücü, 2022). In our study, ratios of n‐nonanal, decenal, and geranyl acetone were lower than those of the study of Ağca et al. (2020). Furthermore, the ratio of β‐ionone was higher than that given in the other study, while the ratio of α‐ionone was compitable. Alcohols such as hexanol and its derivatives, benzyl alcohol and its derivatives, linalool, and terpineol have been reported in tea infusions analyzed by Das et al. (2019). Lee, Chambers, and Chambers IV (2013) almost found linalool and hexanal in all samples by brewing 24 different GTs from 8 different countries. However, 2‐pentene‐1‐ol, 1‐penten‐3‐ol, and benzaldehyde were found in the GT sample collected from Africa. In addition, nonanal was generally found in samples harvested from Southeast Asia.

In our study, 11 compounds found in the GT infusions (i.e., lauryl alcohol, butyl laurate, 5‐hydroxy‐2,4‐decadienoic acid δ‐lactone, davanone B, decyl propionate, 3,7‐dimethyl‐1‐octanol, neodene, isopulegyl acetate, cis‐8‐undecanal, octyl octanoate, and isobornyl benzoate) were determined in the literature. However, there is no literature about the presence of these compounds in GT (Göksu Sürücü, 2022). Moreover, there was no valid literature indicating that tetrahydrofurfuryl butyrate found in GT infusions in our study is naturally found in any other source (Table 2).

Customers can choose from a variety of green teas that reflect diverse harvest times, plant varieties, processing techniques, and growing areas, all of which may contribute to the unique aroma qualities of each tea (Lee, Chambers IV, et al., 2013). In addition to climatic and geographical conditions, production processes can profoundly affect aromatic precursors and the content of glycosidase enzyme, leading to a large variation in the aromatic profile of tea (Choi et al., 2016; Wang et al., 2002; Zheng et al., 2016). Tontul et al. (2013) also reported that the harvest period and shading rate can profoundly affect the aroma profile of two different tea clones in Türkiye (e.g., heptenal, ethyl benzene, tridecane, etc.). Ryu et al. (2012) discovered, using a solid‐phase microextraction (SPME) approach, that teas produced at lower temperatures had fewer volatile chemicals than teas harvested from the same areas at a warmer temperature 1 year later.

One of the most important elements influencing the quality of green tea aroma is processing. According to reports, the overall concentrations of volatile compounds reduced after processing green tea, particularly after the fixing and drying stages, when the temperature was high and may have caused the compounds to evaporate (Cui et al., 2021; Yin et al., 2022). The process by which changes in volatile compounds occur during the spreading of green tea was studied by Qiao et al. (2021). They remarked that a modest spreading might greatly enhance the buildup of aroma volatiles in green tea leaves after harvest.

Brewing conditions are a crucial aspect that can greatly impact the aroma of the tea infusion, which is directly perceived by consumers, after cultivation and processing (Sun et al., 2022). Various brewing variables, including leaf size, temperature, time, water hardness, and brewing apparatus, have been found by numerous researchers to impact the release or formatting of green teas' VOCs (Guo et al., 2019; Sánchez‐López et al., 2020). The sensory qualities and aroma profile of green tea infusion are influenced by brewing settings (temperature, duration of brewing, tea/water ratio, etc.) and brewing water parameters (Cao et al., 2021; Yin et al., 2022). It is remarked that the six primary categories of tea aroma generation mechanisms are: carotenoid derivatives, terpenoid volatiles, glycoside hydrolysates, phenylpropanoids/benzenoids, and products of the Maillard reaction (Wang, Yu, et al., 2021; Yang et al., 2021; Zhang et al., 2020; Zheng et al., 2016; Zhu et al., 2021). The tea infusion's VOCs are diverse, highly dynamic, and comprise many volatiles. In particular, certain varieties of them seem to have a temporal quality since they happen quickly, like while making tea, while others with persistent characteristics are more stable. The information on the quality of the tea's aroma may be lost, resulting in a tea infusion that does not completely reflect the complex aroma profiles. The release, maximizing, and attenuation of aromas are just a few of the numerous temporal changes that occur during the brewing of tea that affect the VOCs (Sánchez‐López et al., 2020; Sun et al., 2022).

Sun et al. (2022) investigated how variations in VOCs affected the headspace and the generation or release of these compounds during the tea‐brewing process. They identified a total of 38 VOCs with headspace (HS)‐SPME/GC–MS, which can be divided into nine types, including six esters, six alcohols, six fatty acids, seven aldehydes, three ketones, two heterocycles, six hydrocarbons, one phenol, and one organosulfide. They identified 22 out of 38 volatiles (57.89%) that changed while the tea was brewing. In our study, extending the brewing time also significantly altered the distribution and amount of aromatic components in GT infusions. For example, the highest number of aroma components was 29 with 25 min of brewing, while the lowest number of aroma components was 19 with 35 min of brewing. Additionally, the theaspirane, a norisprenoid, was altered after 30 min and was not detected in green tea infusions (Table 2). It is indicated that the three elements of the phase equilibrium for the tea–water system are headspace, soluble and insoluble tea solids and water. Stated differently, throughout the tea‐brewing process, the system that includes the VOCs released from the tea leaves into the infusion and the VOCs released from the infusion into the headspace tends to equilibrate. It is also claimed that longer brewing times were more closely associated with a change in the VOC content (Sun et al., 2022). This explanation clarifies the variation in aroma components found in infusions in our study based on brewing time.

Three groups of tea solubles were identified by Long (1979): essentially instantaneous solubles, rapid solubles, and slow solubles, in light of the heterogeneous nature of tea leaves. The instantly and rapidly soluble compounds must be readily reachable to water. Because of their higher molecular weight, the slower‐dissolving components diffuse more slowly through the leaf matrix and into the water or from the inside to the outside. The process that determines the equilibrium rate is said to be the movement of VOCs from the inside of tea leaves over the leaf/water interface (Long, 1977; Sun et al., 2022). In our study, GT1, GT2, and GT3 have different amounts and numbers of VOCs. The most likely elucidation is that, as brewing time increases, components continue to dissolve in tea infusions. This causes changes in concentrations for thermophysical and chemical interactions. Most probably, as the amount of green tea changes, the amount of aroma compounds passing into the infusion also changes and there is a chemical interaction between some aroma compounds. As a result, it is possible that different aroma compounds are formed and some aroma compounds are degraded. This study demonstrated that by regulating the synthesis or degradation of aromatic compounds, the amount of tea used to brew significantly affects the aroma of green teas. The dissolving of the compounds in tea leaves, the duration of time the infusion brewed, the chemical and physical transfer among the GT particles, and the water to create an equilibrium all interacted to produce the volatile performance in the tea infusions. Further elucidation of the biochemical production pathways of the major aromas is necessary to accurately control the aroma components of green tea and more research is needed to investigate this situation.

Due to its widespread use in a variety of environmental mediums, phthalic acid esters (PAEs, i.e. dimethyl phthalate (DMP), diisobutyl phthalate (DiBP), diethyl phthalate (DEP), di‐n‐octyl phthalate (DnOP), di‐n‐butyl phthalate (DnBP), butylbenzyl phthalate (BBP), di‐(2‐ethylhexyl) phthalate (DEHP), etc.) are among the substances that have received the maximum attention from researchers and have generated the maximum number of debates (Li et al., 2022). DEP as a plasticizer was identified in 35 of 36 samples (excluding GT infusion 2 for 60 min). The highest (24.34%) and lowest (9.47%) amounts of DEP were quantified by infusing GT1 for 5 min and GT3 for 55 min, respectively. As well, the existence of DiBP in two GT infusions accompanied by DEP shows the possibility of storing these samples in plastic packaging during storage and transportation. However, the presence of DEP and DiBP infusions can be due to cross‐contamination, possibly from plastic containers (Yamaguchi & Shibamoto, 1981). Diethyl phthalate existence in GT was also reported in the literature (Du et al., 2016; Lo Turco et al., 2015; Lu et al., 2015).

This fact shows that GT may be contaminated after contact with the plastic bags during their storage in the supply warehouse. Another reason may be the preparation and storage conditions of infusions as they were stored in screw‐cap plastic tubes, were wrapped with parafilm, and were kept in a deep freezer at −18°C until the analyses.

## 4 CONCLUSIONS

This study reveals that the amount of GT (5–10 g) and brewing time (5–60 min) have a significant effect on aroma composition, distribution and amount of aromatic constituents in GT. According to the results, the highest aromatic volatile compounds were detected when green tea infusion was prepared by 7.5 g of green tea at 25 min, while the lowest aromatic volatile compounds were detected with 10 min brewing time prepared by 10 g of GT. The most abundant constituents in GT infusions have been determined as phytone (5.67%–12.78%), 2‐decenal (2.33%–11.75%), lauric acid (2.27%–8.19%), methoxy‐1‐methylethyl pyrazine (6.15%–15.83%), α‐ionone (1.88%–11.97%), β‐ionone (4.90%–17.73%), and diethyl phthalate (9.46%–24.32%). Dissolving the compounds in tea leaves and the duration of the infusion during brewing have an effect on these chemicals and their physical transfer from leaves to the tea infusion media.

This comprehensive study on the determination of optimal levels of GT amount and brewing time has importance to enhance desired aromatic volatile compounds, which are important factors influencing worldwide market demand depending on consumers’ acceptance.

## AUTHOR CONTRIBUTIONS

**Canan Göksu Sürücü:** Conceptualization (lead); data curation (lead); formal analysis (lead); funding acquisition (equal); investigation (lead); methodology (lead); resources (equal); writing – original draft (lead); writing – review and editing (lead). **Aysu Tolun:** Investigation (supporting); writing – original draft (supporting); writing – review and editing (supporting). **Ozan Halisçelik:** Methodology (supporting); resources (supporting); writing – review and editing (supporting). **Nevzat Artık:** Funding acquisition (equal); supervision (lead); writing – review and editing (supporting).

## FUNDING INFORMATION

This research did not receive any specific grant from funding agencies in the public, commercial, or not‐for‐profit sectors.

## CONFLICT OF INTEREST STATEMENT

The authors declare no conflict of interest.

## Data Availability

The data that support the findings of this study are available from the corresponding author upon reasonable request.
